# A Single Amino Acid Substitution in the Group 1 *Trypanosoma brucei gambiense* Haptoglobin-Hemoglobin Receptor Abolishes TLF-1 Binding

**DOI:** 10.1371/journal.ppat.1003317

**Published:** 2013-04-18

**Authors:** E. DeJesus, R. Kieft, B. Albright, N. A. Stephens, S. L. Hajduk

**Affiliations:** Department of Biochemistry and Molecular Biology, University of Georgia, Athens, Georgia; University of California, Los Angeles, United States of America

## Abstract

Critical to human innate immunity against African trypanosomes is a minor subclass of human high-density lipoproteins, termed Trypanosome Lytic Factor-1 (TLF-1). This primate-specific molecule binds to a haptoglobin-hemoglobin receptor (HpHbR) on the surface of susceptible trypanosomes, initiating a lytic pathway. Group 1 *Trypanosoma brucei gambiense* causes human African Trypanosomiasis (HAT), escaping TLF-1 killing due to reduced uptake. Previously, we found that group 1 *T. b. gambiense* HpHbR (*Tbg*HpHbR) mRNA levels were greatly reduced and the gene contained substitutions within the open reading frame. Here we show that a single, highly conserved amino acid in the *Tbg*HpHbR ablates high affinity TLF-1 binding and subsequent endocytosis, thus evading TLF-1 killing. In addition, we show that over-expression of *Tbg*HpHbR failed to rescue TLF-1 susceptibility. These findings suggest that the single substitution present in the *Tbg*HpHbR directly contributes to the reduced uptake and resistance to TLF-1 seen in these important human pathogens.

## Author Summary

African trypanosomes are parasites that are able to infect a wide variety of mammals; however, only two sub-species, *Trypanosoma brucei rhodesiense* and *Trypanosoma brucei gambiense*, are able to infect humans. A human innate immune molecule, trypanosome lytic factor-1 (TLF-1), is responsible for this selective protection. TLF-1 killing requires high affinity binding to the trypanosome haptoglobin-hemoglobin receptor (HpHbR), which initiates endocytosis and lysosomal localization of the toxin. *T. b. gambiense* infects humans because it does not bind TLF-1, and several amino acid changes in the HpHbR are conserved in group 1 *T. b. gambiense*. To better understand the mechanism of resistance in these parasites, we analyzed TLF-1 binding to trypanosomes expressing the *T. b. gambiense* HpHbR (*Tbg*HpHbR) and variants in which single amino acids were changed. Our studies showed that a single, highly conserved, amino acid substitution in the *Tbg*HpHbR was sufficient to ablate high affinity TLF-1 binding and contributed to TLF-1 resistance. This likely plays a key role in human infectivity by group 1 *T. b. gambiense*.

## Introduction

Primate specific innate immunity plays a decisive role in defining the host range of African trypanosomes. *Trypanosoma brucei brucei, Trypanosoma congolense* and *Trypanosoma vivax* infect both domesticated and wild mammals but are unable to infect most primates, including humans, because of their susceptibility to two primate specific innate immune complexes, Trypanosome Lytic Factor-1 (TLF-1) and TLF-2 [1]–[3]. TLF-1 and TLF-2, isolated from humans, have similar protein compositions. Both complexes contain apolipoprotein A-1 (apoA-1), a characteristic protein of high-density lipoproteins (HDLs), and two primate-specific proteins, apolipoprotein L-1 (apoL-1) and haptoglobin related protein (Hpr) [3]–[8]. Despite similarities in protein composition, the two complexes differ significantly, TLF-2 containing IgM and having little associated lipid, while TLF-1 is a minor sub-class of HDL (ρ = 1.21–1.26 g/ml) which is ∼40% lipid by mass [9].

TLF-1 killing of *T. b. brucei* requires high affinity binding within the flagellar pocket, a specialized region of the trypanosome cell surface, followed by endocytosis and lysosomal localization [10]. Within the acidic lysosome, TLF-1 is activated leading to disruption of the lysosome and cell lysis [5], [10]–[13]. Critical to initiating the lytic pathway is the binding of TLF-1 to the *T. b. brucei* haptoglobin-hemoglobin receptor (*Tbb*HpHbR) [11], [14], [15]. Haptoglobin (Hp) is an acute phase protein produced at high levels in all mammals, which binds and detoxifies free hemoglobin (Hb) by facilitating its clearance from the circulation [15]. Since African trypanosomes are heme auxotrophs, *Tbb*HpHbR has been proposed to function as a nutrient receptor providing heme to these parasites [14]. Unlike the mammalian HpHb scavenger receptor (CD163) the *Tbb*HpHbR also binds Hpr present in TLF-1 when complexed with Hb [16].

Two mechanisms of trypanosome resistance to TLF-1, and therefore human infectivity, have been described [17]. *Trypanosoma brucei rhodesiense*, the cause of acute human African trypanosomiasis (HAT), has evolved the human serum resistance associated protein (SRA), which binds and neutralizes TLF-1 killing [16]–[19]. A member of the variant specific glycoprotein (VSG) family, SRA, is a glycophosphatidylinositol-anchored protein that is synthesized in the endoplasmic reticulum and transiently presented on the surface of the trypanosome within the flagellar pocket. However, its steady state distribution suggests it is rapidly endocytosed and localizes predominately to endosomes in *T. b. rhodesiense* [20]–[22]. SRA tightly binds the apoL-1 component of TLF-1, providing complete protection against TLF-1 killing [5]. It is assumed that SRA also binds apoL-1 in TLF-2 and inhibits its activity.

*Trypanosoma brucei gambiense*, the causative agent of chronic HAT, lacks SRA. We recently reported that expression of the *T. b. gambiense* HpHbR (*Tbg*HpHbR) was reduced in the group 1 subtype of *T. b. gambiense*, suggesting that decreased expression of the receptor contributed to TLF-1 resistance and human infectivity [23]. We also observed that the *Tbg*HpHbR gene, from four distinct geographic isolates of group 1 *T. b. gambiense*, contained four non-synonymous amino acid substitutions within the coding sequence for the mature protein [23]. A more extensive analysis of a large number of isolates further revealed a single leucine (L) to serine (S) substitution, at amino acid 210 of *Tbb*HpHbR which was conserved in all group 1 *T. b. gambiense* isolates examined [24]. This led to the suggestion that this substitution might reduce the affinity of *Tbg*HpHbR for TLF-1 [23]–[25]. Recently, the crystal structure of the HpHbR was deduced allowing new structure-function analysis. A domain of HpHbR, protruding beyond the VSG coat, possesses a hydrophobic core in which ligand binding is predicted to occur. The L210S substitution present in *T. b. gambiense* was predicted to disrupt the core of the head structure of HpHbR thus eliminating ligand binding [26]. Other mechanisms of resistance to TLF-1 and human serum must exist since group 2 *T. b. gambiense* lacks SRA yet expresses the functional HpHbR and takes up TLF-1 [24]. The mechanism of group 2 *T. b. gambiense* resistance to TLF-1 remains unknown.

In the studies reported here, we developed a *T. b. brucei* double knockout line (*Tbb*HpHbR^−/−^) to directly test the function of each of the four non-synonymous amino acid substitutions in the *Tbg*HpHbR. Expression of *Tbg*HpHbR in the *Tbb*HpHbR^−/−^ knockouts did not restore TLF-1 binding or killing. However, the substitution of serine for leucine, at position 210, restored high affinity TLF-1 binding and susceptibility. Changes to the other three substituted amino acids in the *Tbg*HpHbR had no effect on TLF-1 binding, uptake or killing. These results, together with our previous finding, indicate that TLF-1 resistance has exerted strong selective pressure on group 1 *T. b. gambiense*, resulting both in decreased expression levels and loss of function substitutions in the *Tbg*HpHbR.

## Methods

### Trypanosomes, growth and transfections

Bloodstream form *T. b. brucei* Lister 427(MiTat 1.2) were grown at 37°C under 5% CO_2_ in HMI-9 medium supplemented with 10% fetal bovine serum (Sigma-Aldrich) and 10% Serum-Plus (Sigma-Aldrich). HpHbR KO constructs were generated after cloning HpHbR flanking sequences onto blasticidin and hygromycin resistance genes [23]. All primers used in the studies reported here are listed in Table S1. 3×10^7^/ml trypanosomes were transfected with 5 µg of NotI digested DNA using the Amaxa nucleofection system according to the manufacturer's instructions. (Human T Cell Nucleofactor Kit, program X-001). Transfected cells were then allowed to recover for 24 hours before addition of blasticidin (2.5 µg/ml) or hygromycin (2.5 µg/ml). Cell lines were clonally selected prior to a second round of transfection. To obtain HpHbR double knockout cell line, we transfected the second HpHbR drug resistance construct into the single allele *Tbb*HpHbR^+/−^ lines. To examine the effects of amino acid substitution on HpHbR function, stable cell lines expressing ectopic copies of the *Tbb*HpHbR, *Tbg*HpHbR or the individual *Tbg*HpHbR substitutions were prepared by targeting to the tubulin locus and selection with phleomycin (2.5 µg/ml) [23]. To determine growth rates, cells were grown to mid-log phase and diluted to 1×10^4^/ml. Cell counts, determined by hemocytometer, were carried out until stationary phase. Growth curve data is in triplicate.

### Epitope tagging of HpHbR

An HA-epitope tag was cloned into the *Tbg*HpHbR construct via a three-step PCR method. The HA-tag was added downstream of the signal peptide. Once completed, the construct was sequenced and digested with NotI and ApaI (5 µg total) prior to transfection. Transfections and cloning were carried out as described above.

### *Tbg*HpHbR mutant cell lines

The construct used to generate the *Tbg*HpHbR cell line [23] was subjected to site-directed mutagenesis to generate the four *Tbg*HpHbR substitutions of S210L, V293A and GA369-370EG. Mutagenesis was carried out according to manufactures instructions (Agilent Technologies). *Tbb*HpHbR^−/−^ cells were transfected independently with the mutagenized constructs. Transfections and cloning were carried out as described above. Mutant *Tbg*HpHbR constructs were sequenced with HpHbR sequence primers (sense and antisense). To prepare an HpHbR over-expressing cell line, PCR products were generated with Platinum High Fidelity Taq Polymerase (Invitrogen), gel purified, digested with EcoRI and cloned into the pURAN over-expression constructs [27]. Prior to transfection into *Tbb*HpHbR^−/−^ cells, pURAN HpHbR constructs were linearized with BstXI. Both strands were sequenced with HpHbR sequence primers (sense and antisense).

### TLF-1 purification, lytic activity and binding

TLF-1 purification, labeling and survival assays were performed as previously described [28]. Briefly, for the survival assays, trypanosomes were harvested from mid-log phase cultures, washed and re-suspended at a final concentration of 1×10^6^/ml in complete HMI-9 media. Susceptibility to hemoglobin (Hb) bound TLF-1 was determined over a range of TLF-1 concentrations following incubation at 37°C for 16 hours. The number of surviving cells was determined by hemocytometer count with phase contrast microscopy. All survival assays were done in triplicate.

### Southern analysis

For Southern analysis, 5 µg genomic DNA was digested with EcoRI. DNA was fractionated on a 0.6% agarose gel and transferred to a nitrocellulose membrane (Amersham Hybond-N^+^). Pre-hybridization was with a solution containing 40% formamide (Sigma), 3× SSC, 10× Denhardt's, 20 µg/ml salmon sperm DNA, 5% dextran sulfate and 2% SDS at 42°C for three hours. The P^32^ labeled probe, specific for the region upstream of the 5′-flanking regions was added to the hybridization mix and incubated at 42°C overnight. The probe sequence is listed in Table S1. Blots were then washed in a solution containing 3× SSC/0.1% SDS at 55°C for 30 minutes then a final stringency of 0.3× SSC/0.1% SDS at 65°C for 20 minutes. Blots were exposed to a storage phosphor screen (Molecular Dynamics) and analyzed on a STORM-860 PhosphorImager (GE Healthcare).

### TLF-1 binding and uptake studies

All TLF-1 binding and uptake studies were carried-out with Alexa-Fluor 488 TLF-1 that was labeled according to manufacture instructions (Invitrogen). Alexa-488 TLF-1 was incubated with an excess of Hb for 10 minutes on ice prior to analysis of binding. The binding and uptake of Alexa-488 TLF-1 was examined by either fluorescence microscopy or FAC analysis. To measure the amount of binding by fluorescence microscopy, the fluorescence intensity values from AxioVision v4.6 software (www.zeiss.com) was plotted versus TLF-1 concentrations. Imaging was carried out using a Zeiss Axio Observer inverted microscope. Quantification of Alexa-488 TLF-1 was done on compressed images.

To measure TLF-1 uptake by FAC analysis, cells were grown to mid-log phase, collected, washed and resuspended (1×10^7^/ml) in HMI-9 supplemented with 1% bovine serum albumin (BSA), 1% glucose. Alexa-488 TLF-1, and excess Hb, were added to the cells followed by incubation at 37°C for 30 minutes. Uptake was stopped by placing the tubes on ice followed by two washes with ice-cold phosphate buffered saline buffer (PBS) (10 mM NaP_i_, 137 mM NaCl, pH 7.4). The amount of TLF-1 uptake was determined using Cyan cytometer (DAKO) and FlowJo software. Uptake was also measured by fluorescence microscopy. Following incubation, cells were washed two times with ice cold PBS. Following the washes, cells were spread onto glass slides, methanol-fixed for 5 min, at −20°C, and analyzed by fluorescence microscopy. Images were captured with the same exposure and were contrasted to the same extent. To analyze only binding in the flagellar pocket, pre-chilled Alexa-488 TLF-1/Hb complexes were added to cells in ice-cold HMI-9 supplemented with 1% BSA, 1% glucose and further incubated at 3°C for two hours. Cells were washed two times with ice-cold PBS, kept on ice and subjected to FAC analysis. All binding and uptake experiments analyzed by FAC analysis were done in triplicate. For fluorescence microscopy, approximately 100 cells per data point were analyzed in triplicate. All binding data collected were analyzed using Graphpad Prism software, version 4.0a. To better localize the distribution of TLF-1 binding to the flagellar pocket, PFA fixed and methanol treated trypanosomes were incubated with a mouse anti-paraflagellar rod (PFR) antibody (generously provided by Dr. Diane McMahon-Pratt, New Haven) at a dilution of 1∶1,000 followed by a secondary antibody staining with Alexa Fluor 594 mouse IgG (Invitrogen). Serial image z-stacks were acquired through oil immersion optics with exposure times kept constant for each experiment. Imaging was carried out using a Zeiss Axio Observer inverted microscope equipped with an AxioCam HSm camera and analyzed with the AxioVision v4.6 software (Zeiss). A single stack is shown for each experiment, with individual channels contrasted to the same extent for each image set.

### Competition binding studies

Specificity of TLF-1 binding to trypanosomes was analyzed using competition-binding studies with the unlabelled TLF-1 and Hp 1-1. Cells were collected, washed and resuspended (1×10^7^/ml) in ice-cold HMI-9 supplemented with 1% BSA, 1% glucose then transferred to 3°C for at least 10 minutes. Alexa-488 conjugated TLF-1 (3 nM constant) was complexed with hemoglobin (50 nM) at 4°C for 10 minutes. Increasing concentrations of unlabeled competitor were incubated with Hb (50 nM) for 10 minutes at 4°C. Competing ligands were then mixed with the Alexa-488 conjugated TLF-1/Hb, added to cells at 3°C and allowed to incubate for two hours. Cells were then transferred to ice, washed with ice-cold 1× PBS and analyzed by Cyan cytometer and FlowJo software. For studies without Hb, competitors were added to Alexa-488 TLF-1/Hb (6 nM) in the same increasing molar concentrations and taken through the same protocol as previously described. All competition studies were done in triplicate.

### RT-PCR of expressed HpHbR and qPCR

Total RNA was isolated with Tripure Isolation Reagent (Roche). cDNA was generated in a Reverse Transcription (RT) reaction (Promega). Control reactions were performed with enolase, as well as reactions without added RT. Real time PCR was performed with and iCycler (iQ5 multicolor real-time PCR detection system; Bio-Rad) using cDNA from an equivalent of 20 ng of total RNA, 6 pmol sense primer, 6 pmol antisense primer, 10 µl SYBR green PCR master mix (Fermentas) in a final volume of 20 µl. Real time PCR conditions were: one cycle of 95°C for 3 min, followed by 40 cycles of 95°C for 15 s, 60°C for 30 s and 72°C for 30 s. The relative amounts of specific cDNA between samples were calculated using CT values calculated with the iQ5 optical detection system software. All qRT-PCR reactions were carried out with a splice leader RNA sense primer and gene specific anti-sense primers. Triplicate analyses were performed for each parasite line. All primers were designed using Integrated DNA Technologies software.

### Immunoblotting analysis

For western analysis of HA-tagged HpHbR, total cellular protein was prepared from *Tbb*HpHbR^−/−^, Rab5a^HA^ and *Tbg*HpHbR^HA^ cell lines and and analyzed, based on cell equivalents, as previously described [22]. Rat monoclonal anti-HA–biotin (Roche Diagnostics, Indianapolis, IN) was used at a dilution of 1∶1000, with streptavidin-HRP conjugate (Invitrogen, Camarillo, CA) used for secondary detection at 1∶5000.

## Results

### Generation of an HpHbR^−/−^ cell line

Previously, we described the isolation of a TLF-1 resistant line of *T. b. brucei* following *in vitro* selection for growth in the presence of human HDLs [23], [29]. The resistance phenotype correlated with reduced expression of the *Tbb*HpHbR, susceptibility being restored by ectopic expression of the *Tbb*HpHbR from a different chromosomal locus. We also observed that ectopic expression of the *Tbg*HpHbR failed to restore TLF-1 uptake or susceptibility, suggesting that substitutions to the *Tbg*HpHbR might contribute to TLF-1 resistance in this important human pathogen [23]. Initial sequence analysis of four *T. b*. *gambiense* isolates revealed five non-synonymous amino acid substitutions, four in the coding sequences of the mature protein, when compared to *Tbb*HpHbR [23]. To test whether these substitutions lead to loss of TLF-1 binding, we generated a *T. b. brucei* HpHbR^−/−^ knockout cell line and then systematically tested the ability of each of the four substitutions to restore TLF-1 binding to the *Tbg*HpHbR.

**Figure 1:**
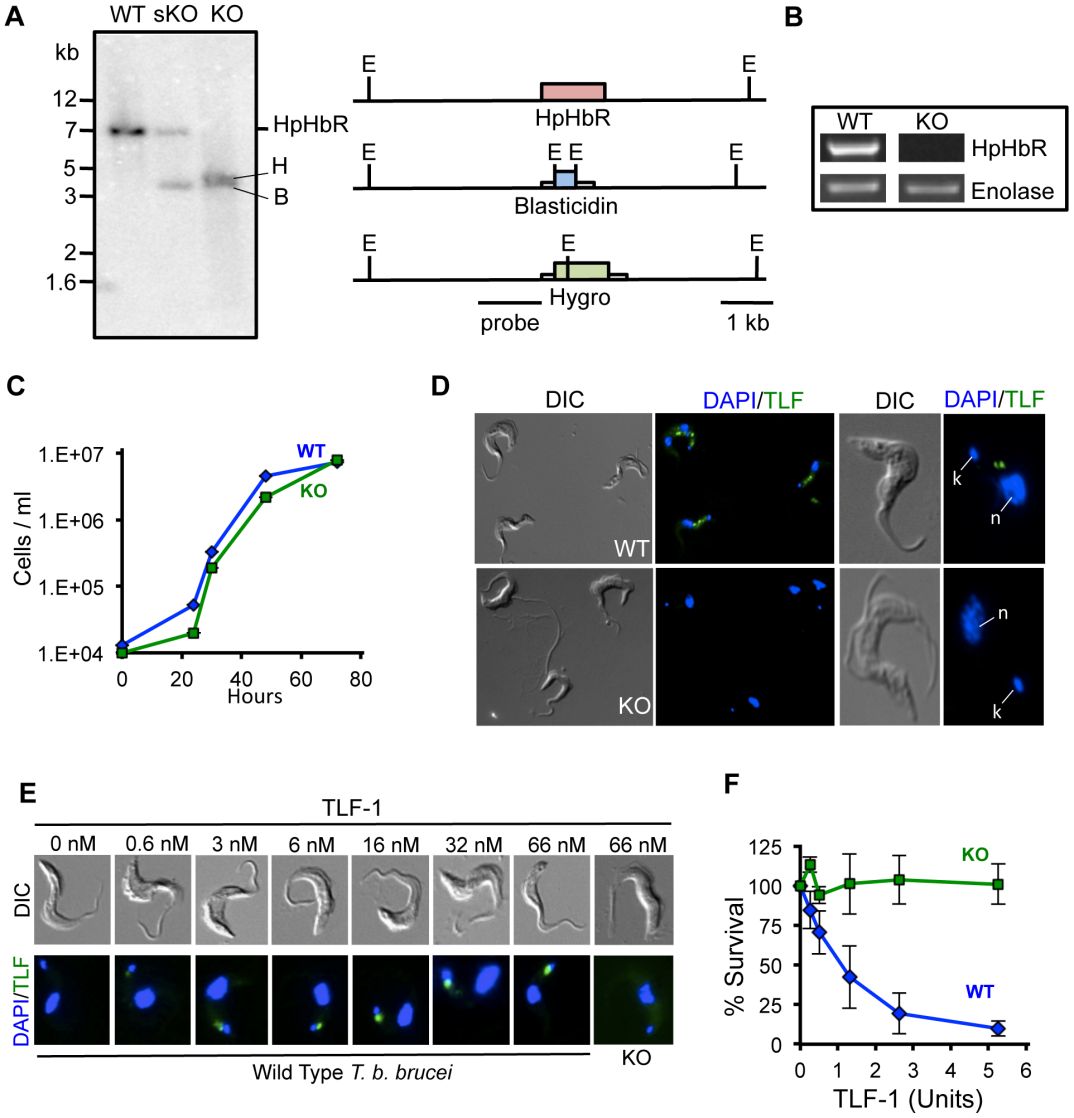
Generation of *Tbb*HpHbR^−/−−/−^ cells. (**A**) Both allelic copies of the *Tbb*HpHbR were replaced by homologous recombination with the blasticidin (B) and hygromycin (H) resistance cassettes. Southern analysis was carried out with DNA from *T. b. brucei* (WT), blasticidin HpHbR^+/−^ single knockout line (sKO) of the *Tbb*HpHbR and a line with both alleles replaced (KO). Because of internal EcoRI (E) sites in the drug resistance constructs, fragments of 3.9 kb and 4.2 kb are generated in contrast to the 7 kb *Tbb*HpHbR in WT cells. (**B**) Expression levels of HpHbR mRNA were determined by RT-PCR from WT and KO cells. Enolase was used as the loading control. (**C**) *In vitro* growth of WT and KO cell lines at 37°C. (**D**) DIC and fluorescence microscopy of the WT and KO cell lines after incubation with Alexa-488 TLF-1 (37°C for 30 minutes). Higher magnification images (right side of panel) the position of the kinetoplast (k), and nucleus (n) was visualized by DAPI staining. (**E**) *T. b. brucei* WT cells were incubated with Alexa-488 TLF-1 for 1 hour at 3°C over a range of 0 to 66 nM. Binding was localized to the flagellar pocket and was concentration dependent. (**F**) Susceptibility of *T. b. brucei* (WT) and *Tbb*HpHbR^−/−^ cells (KO) to TLF-1 killing was determined following a 16 hour incubation at 37°C. In these studies a TLF-1 killing unit = 0.019 nM.

The HpHbR knockout cell lines were prepared in *T. b. brucei* 427-221 (Lister 427) cells by sequentially replacing the complete coding sequence for each *Tbb*HpHbR allele with the coding sequences for hygromycin and/or blasticidin (Figure 1A). Replacement of the coding sequence for *Tbb*HpHbR, in both single *Tbb*HpHbR^+/−^ (sKO) and double *Tbb*HpHbR^−/−^ (KO) knockouts, was verified by Southern blot hybridization of genomic DNA digested with EcoRI. The expected size restriction fragments were detected when blots were hybridized with a probe complementary to the 5′ flanking sequence of *Tbb*HpHbR. A 7.0 kb fragment was detected in untransfected *Tbb*HpHbR while EcoRI sites in both the blasticidin and hygromycin gene constructs gave rise to smaller fragments (3.9 kb and 4.2 kb respectively) (Figure 1A). PCR analysis of genomic DNA from WT and *Tbb*HpHbR^−/−^ cells, with oligonucleotide probes complementary to coding sequences in the *Tbb*HpHbR, showed that the *Tbb*HpHbR gene had been deleted in the *Tbb*HpHbR^−/−^ cells (Figure S1). Furthermore, (q)RT-PCR with total RNA from WT *T. b. brucei* and *Tbb*HpHbR*^−/−^* showed that double knockout cells do not express *Tbb*HpHbR (Figure 1B, Table S2). The generation of a stable *Tbb*HpHbR^−/−^ cell line showed that this gene was non-essential in the bloodstream stage of *T. b. brucei* used in these studies. In addition, only a very slight reduction in growth rate was observed (Figure 1C).

### *Tbb*HpHbR is necessary for TLF-1 binding, uptake and killing

We examined whether the *Tbb*HpHbR^−/−^ cells were deficient in TLF-1 uptake. When WT *T. b. brucei* was incubated at 37°C with Alexa-488 conjugated TLF-1 (10 nM), cells rapidly accumulate TLF-1 in endosomes and lysosomes, whereas no detectable Alexa-488 TLF-1 internalization was observed in *Tbb*HpHbR^−/−^ cells (KO) (Figure 1D). As previously reported, uptake of TLF-1 was dependent on the addition of Hb prior to incubation with trypanosomes [28]. Additionally, it was shown that at 3°C, Alexa-488 TLF-1 localized specifically to the flagellar pocket [22]. To determine whether TLF-1 uptake at this low concentration was dependent on flagellar pocket binding, WT and *Tbb*HpHbR^−/−^ cells were incubated at 3°C with Alexa-488 TLF-1 (Figure 1E). At concentrations as low as 0.6 nM, TLF-1 binding to the flagellar pocket was detectable and was concentration dependent up to 66 nM. No TLF-1 binding to *Tbb*HpHbR^−/−^ cells was observed at concentrations up to 66 nM (Figure 1E). In addition, *Tbb*HpHbR^−/−^ cells were highly refractory to TLF-1 killing at concentrations of 0.1 nM (Figure 1F). These studies showed that the *Tbb*HpHbR was required for high affinity TLF-1 binding and further supports the role of this receptor in trypanosome killing.

### Functional analysis of the *Tbg*HpHbR

**Figure 2:**
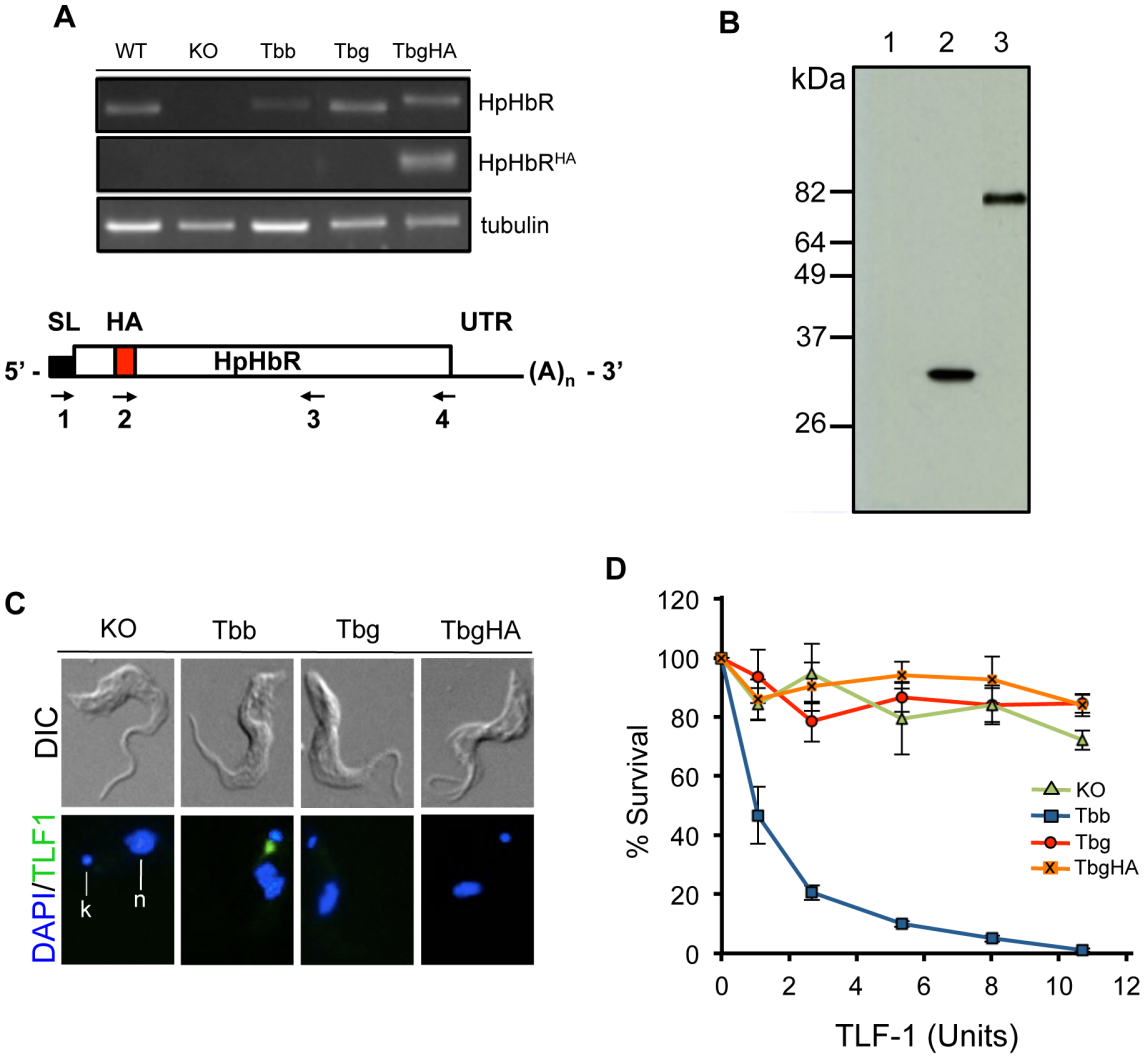
Generation and functional analysis of the HpHbR in *Tbb*HpHbR^−/−−/−^ cells. (**A**) Expression levels of HpHbR mRNA were determined by RT-PCR from wild type *T. b. brucei* (WT), *Tbb*HpHbR^−/−^ (KO) cells ectopically expressing the *Tbb*HpHbR (Tbb), *Tbg*HpHbR (Tbg) and *Tbg*HpHbR^HA^ (TbgHA) genes. β-tubulin used as the loading control. Schematic of HA-epitope tagged HpHbR mRNA showing the primer binding sites for full length HpHbR mRNA (primers 1 and 4) and HA-tagged HpHbR mRNAs (primers 2 and 3) (**B**) Western analysis of KO, Rab5a^HA^ and TbgHA showing expression of the *Tbg*HpHbR. 1: *Tbb*HpHbR^−/−^, 2: Rab5a^HA^, 3: *Tbg*HpHbR^HA^ (**C**) Fluorescence microscopy of Alexa-488 TLF-1 binding to *Tbb*HpHbR^−/−^ (KO), *Tbb*HpHbR (Tbb), *Tbg*HpHbR (Tbg) and *Tbg*HpHbR^HA^ (TbgHA). Kinetoplast (k), Nucleus (n). (**D**) Susceptibility of Tbb, Tbg, TbgHA and KO cells to TLF-1 killing determined by a 16 hour incubation at 37°C. In these studies a TLF-1 killing unit = 0.86 nM.

In order to determine whether the *Tbg*HpHbR was functional in TLF-1 binding and subsequent killing, stable cell lines, ectopically expressing the *Tbb*HpHbR and *Tbg*HpHbR genes, were prepared in the *Tbb*HpHbR^−/−^ background. In addition, to verify HpHbR expression, an HA-epitope tagged variant of the *Tbg*HpHbR (*Tbg*HpHbR^HA^), in the *Tbb*HpHbR^−/−^ background, was also prepared (Figure 2A). Expression of *Tbb*HpHbR, *Tbg*HpHbR and *Tbg*HpHbR^HA^ was determined by nested RT-PCR allowing detection of both endogenous and HA-tagged HpHbR mRNAs (Figure 2A). The level of HpHbR mRNA was comparable in all cell lines (Table S2). The expression of *Tbg*HpHbR^HA^ was also evaluated by western blot with antibodies specific to the HA-tagged HpHbR (Figure 2B). A single band, migrating around 80 kDa, was visible in *Tbg*HpHbR^HA^ cells, but not in the *Tbb*HpHbR^−/−^ cell line (Figure 2B). Specificity of the anti-HA antibody was verified with a cell line expressing a HA-tagged Rab5A (Figure 2B) [22]. To determine whether the *Tbg*HpHbR^HA^ was functional in TLF-1 binding and uptake, cells were examined for TLF-1 binding and subsequent killing (Figures 2C and 2D). *Tbb*HpHbR, but not *Tbg*HpHbR or *Tbg*HpHbR^HA^, restored both TLF-1 binding and killing in the *Tbb*HpHbR^−/−^ background (Figures 2C and 2D). We also prepared a HA-epitope tagged variant of the *Tbb*HpHbR (*Tbb*HpHbR^HA^), in the *Tbb*HpHbR^−/−^ background. However, perhaps due to the positioning of the HA-epitope within the highly structured cytosolic domain of the receptor, no detectable signal was seen on western blots (unpublished data). It is unlikely that the *Tbb*HpHbR^HA^ was not expressed since mRNA levels were comparable to the other receptor knock-in lines and TLF-1 binding and susceptibility were restored to wild type levels in these cells. Together these results indicate that *Tbb*HpHbR retains the ability to bind TLF-1 while *Tbg*HpHbR and *Tbg*HpHbR^HA^, while expressed at comparable levels, do not function in TLF-1 binding, uptake or trypanosome killing.

### Functional analysis of sequence polymorphisms in the *Tbg*HpHbR

**Figure 3:**
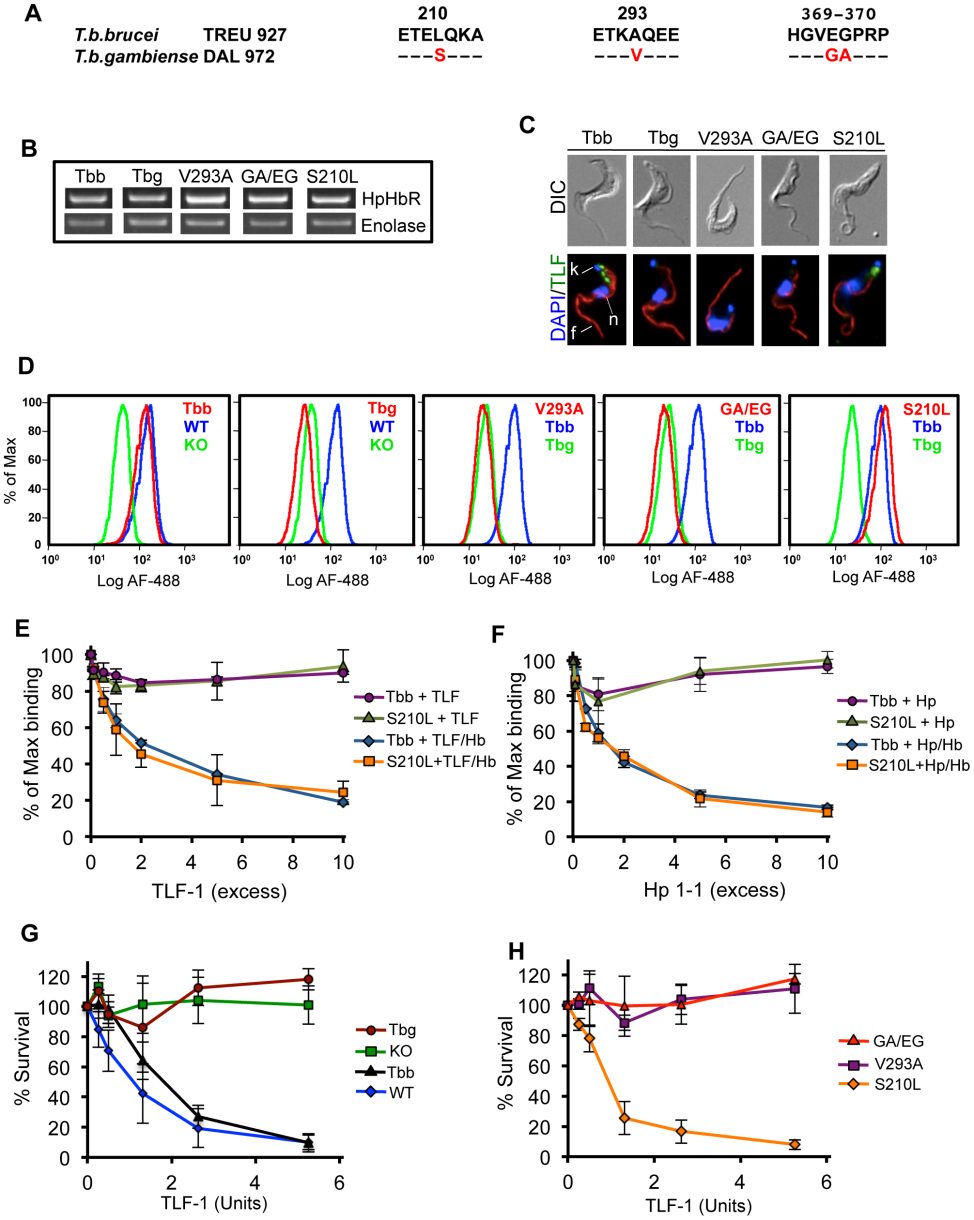
Effects of amino acids substitutions in *Tbg*HpHbR on TLF-1 uptake and killing. (**A**) Sequence alignment of *T. b. brucei* and *T. b. gambiense* HpHbR. Four amino acids in the *Tbg*HpHbR, positions 210, 293 and 369–370, within the mature coding sequence. (**B**) Expression levels of HpHbR mRNA were determined by RT-PCR from *Tbb*HpHbR^−/−^ cells ectopically expressing the *Tbb*HpHbR (Tbb), *Tbg*HpHbR (Tbg), *Tbg*HpHbR^V293A^ (V293A), *Tbg*HpHbR^GA369/370EG^ (GA/EG) and *Tbg*HpHbR^S210L^ (S210L) genes. (**C**) Fluorescence microscopy of the *Tbb*HpHbR^−/−^ cells ectopically expressing the Tbb, Tbg, V293A, GA/EG and S210L genes after incubation with 20 nM Alexa-488 TLF-1 at 37°C for 30 minutes. Kinetoplast (k), Nucleus (n), Flagellum (f). (**D**) FAC analysis of Alexa-488 TLF-1 uptake by wild type *T. b. brucei* (WT), *Tbb*HpHbR^−/−^ (KO), and the *Tbb*HpHbR^−/−^ cell lines ectopically expressing the Tbb, Tbg, V293A, GA/EG and S210L genes. (**E**) Competition for Alexa-488 TLF-1 binding with unlabeled TLF-1 to *Tbb*HpHbR^−/−^ cells ectopically expressing the Tbb and S210L (plus and minus added Hb). (**F**) Competition for Alexa-488 TLF-1 binding with unlabeled Hp 1-1 to *Tbb*HpHbR^−/−^ cells ectopically expressing the Tbb and S210L gene (plus and minus added Hb). (**G**) Susceptibility to TLF-1 killing was determined based on *in vitro* TLF-1 survival assay (16 hour) for WT and *Tbb*HpHbR^−/−^ cells ectopically the Tbb and Tbg genes. (**H**) Susceptibility to TLF-1 was determined for V293A, GA/EG and S210L by an *in vitro* TLF-1 survival assay (16 hour). In these studies a TLF-1 killing unit = 0.019 nM.

Sequence analysis of a small number of isolates of group 1 and 2 *T. b. gambiense*, *T. b. rhodesiense* and *T. b. brucei* led to the initial hypothesis that a limited number of amino acid substitutions may contribute to reduced uptake of TLF-1 by cells expressing the *Tbg*HpHbR [23] (Figure 3A). In a more comprehensive geographic and taxonomic analysis of HpHbR sequences, a single substitution replacing a leucine with a serine at position 210 was observed in all group 1 *T. b. gambiense* and was not observed in TLF-1 susceptible *T. b. brucei* or in *T. b. rhodesiense* [24]. To test whether any of the substitutions in the *Tbg*HpHbR could individually restore TLF-1 binding and killing, each of the *T. b. gambiense* specific amino acid substitutions were changed back to the amino acid found in the *Tbb*HpHbR (Figure 3A). The steady state levels of *Tbb*HpHbR, *Tbg*HpHbR, *Tbg*HpHbR^S210L^, *Tbg*HpHbR^V293A^ and *Tbg*HpHbR^GA369-370EG^ mRNA s were evaluated by (q)RT-PCR (Figure 3B, Table S2). The levels of expression of these ectopically expressed genes were comparable in all five analyzed cell lines.

In order to determine whether amino acid changes in the *Tbg*HpHbR affected TLF-1 uptake, each cell line was examined by fluorescence microscopy and flow cytometry following incubation with Alexa-488 TLF-1 for 30 minutes at 37°C (Figure 3C and 3D). Fluorescence microscopy showed that *Tbb*HpHbR expressing cells endocytosed TLF-1 and that most was localized to the posterior region of the cells between the kinetoplast and nucleus consistent with lysosomal trafficking (Figure 3C). Flow cytometry indicated that the amount of TLF-1 taken up by the *Tbb*HpHbR cells was similar to that seen in WT *T. b. brucei* (Figure 3D). In contrast, *Tbg*HpHbR cells showed no detectable uptake of TLF-1 either by fluorescence microscopy or flow cytometry analysis (Figure 3C and 3D). Similarly, *Tbg*HpHbR^V293A^ and *Tbg*HpHbR^GA369-370EG^ did not take-up TLF-1 and appeared identical to *Tbg*HpHbR cells. However, the single amino acid change at position 210 of the *Tbg*HpHbR, from serine to leucine, restored TLF-1 uptake and localization to levels seen in the *Tbb*HpHbR cells (Figure 3C and 3D). The specificity of TLF-1 binding in *Tbb*HpHbR and *Tbg*HpHbR^S210L^ was examined by competition binding studies with unlabeled TLF-1 or Hp1-1 in the presence or absence of Hb (Figure 3E and 3F, respectively). When complexed with Hb both unlabeled Hp 1-1 and TLF-1 effectively competed for TLF-1 binding. These results indicated that the HpHbR mediated all TLF-1 binding in these cells.

To determine whether susceptibility to TLF-1 killing was also influenced by the changes to the HpHbR, cell lines expressing *Tbb*HpHbR, *Tbg*HpHbR, *Tbg*HpHbR^S210L^, *Tbg*HpHbR^V293A^ and *Tbg*HpHbR^GA369-370EG^ were incubated with increasing concentrations of TLF-1 and the percentage of cells surviving after 16 hours was determined (Figure 3G and 3H). As expected, based on uptake studies, cells expressing *Tbb*HpHbR and *Tbg*HpHbR^S210L^ were fully susceptible to TLF-1. *Tbg*HpHbR, *Tbg*HpHbR^V293A^ and *Tbg*HpHbR^ GA369-370EG^ were resistant to TLF-1 killing. Together these studies show that the single amino acid change of serine to leucine at position 210 of *Tbg*HpHbR is sufficient to restore both TLF-1 uptake and killing to levels seen in cells expressing the *Tbb*HpHbR. This finding is consistent with the substitution to the HpHbR in *T. b. gambiense* playing a critical role in human infectivity.

### TLF-1 binding affinities of cells expressing variant HpHbR

**Figure 4:**
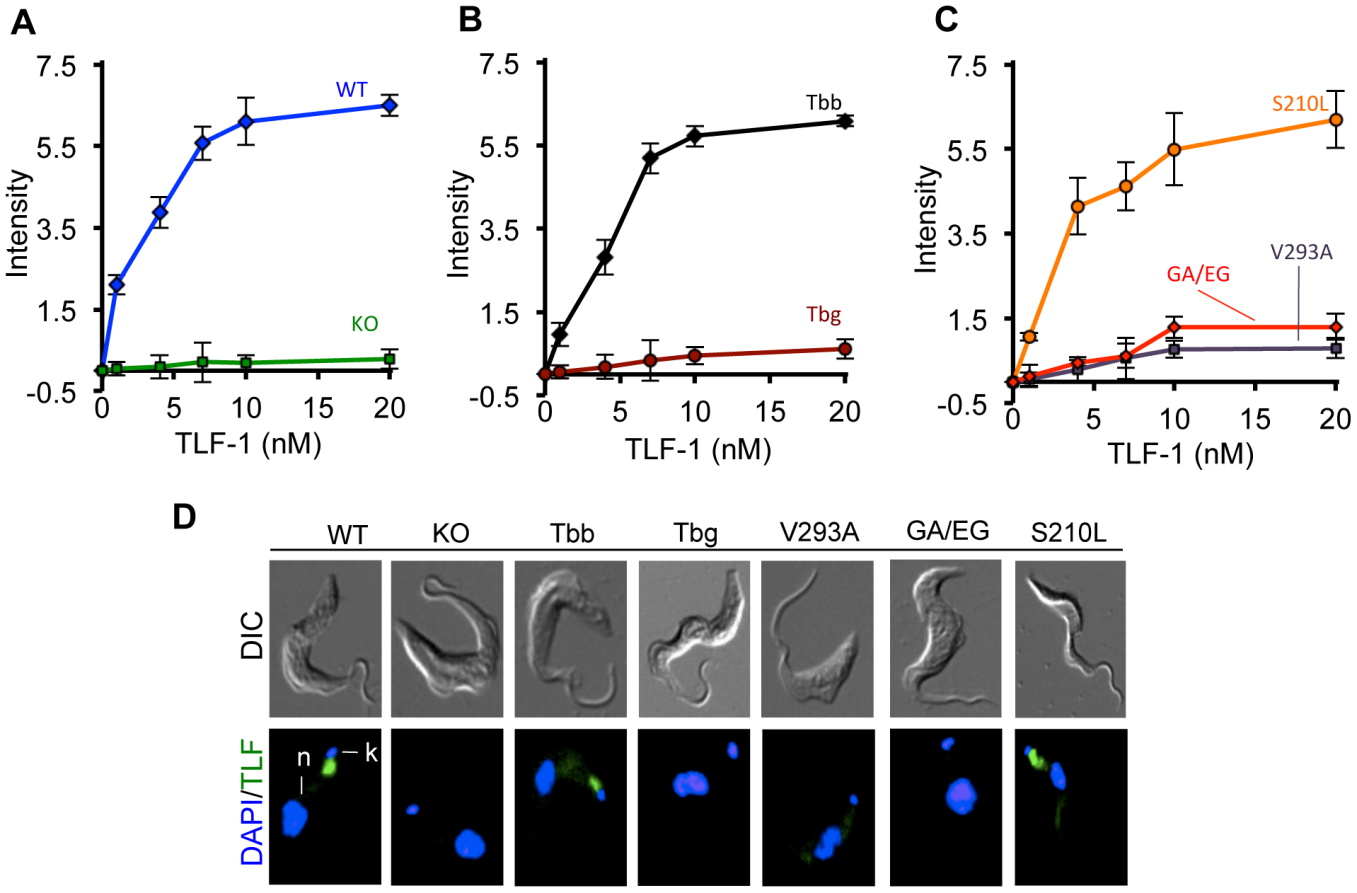
Saturation binding studies with TLF-1. (**A**) Binding affinities were estimated based on half-maximal binding at 3°C with varying concentrations of Alexa-488 TLF-1. Binding to *T. b. brucei* (WT) and *Tbb*HpHbR^−/−^ (KO); (**B**) *Tbb*HpHbR^−/−^ cells ectopically, *Tbb*HpHbR (Tbb) and *Tbg*HpHbR (Tbg); and (**C**) *Tbb*HpHbR^−/−^ cells ectopically expressing *Tbg*HpHbR^V293A^ (V293A), *Tbg*HpHbR^GA369/370EG^ (GA/EG) and *Tbg*HpHbR^S210L^ (S210L). Affinities for the Alexa-488 TLF-1 with WT were Kd = 3.96±0.31 nM, Tbb Kd = 4.12±0.25 nM, S210L Kd = 3.96±0.35 nM. No binding was observed for Tbg, KO, V293A or GA/EG cell lines. (**D**) Fluorescence microscopy showed that Alexa-488 TLF-1 binding was seen in the flagellar pocket of WT, Tbb and S210L. Kinetoplast (k), Nucleus (n).

To evaluate the effect of the amino acid substitutions in the *Tbg*HpHbR on the binding affinity for TLF-1, a live cell-binding assay was developed with Alexa-488 TLF-1. Cells were incubated at 3°C for 2 hours with varying concentrations of Alexa-488 TLF-1. Unbound TLF-1 was removed by washing in ice-cold 1× PBS and the amount and location of TLF-1 binding evaluated by FAC analysis and fluorescence microscopy (Figure 4, Figure S3). Alexa-488 TLF-1 localized exclusively to the flagellar pocket (Figure 4D) and cell associated fluorescence was concentration dependent in WT *T. b. brucei*, *Tbb*HpHbR and *Tbg*HpHbR^S210L^ cell lines (Figure 4A–C). No detectable TLF-1 binding was seen in *Tbb*HpHbR^−/−^, *Tbg*HpHbR, *Tbg*HpHbR^V293A^, and *Tbg*HpHbR^GA369-370EG^ cell lines (Figure 4C and D). To determine whether the affinity for TLF-1 differed in the *Tbb*HpHbR^−/−^ cell lines expressing *Tbb*HpHbR, *Tbg*HpHbR, *Tbg*HpHbR^S210L^, *Tbg*HpHbR ^V293A^ and *Tbg*HpHbR^ GA369-370EG^ we performed saturation binding studies with Alexa-488 TLF-1 (Figure 4A–C). The binding affinity was estimated based on half-maximal binding. Both WT *T. b. brucei* and *Tbb*HpHbR had high affinity for TLF-1 (3.96±0.31 nM and 4.12±0.25 nM, respectively). The *Tbg*HpHbR^S210L^ cells also bound TLF-1 with similar affinity (3.96±0.35 nM). Consistent with the results obtained by microscopic analysis, TLF-1 binding was not observed in the *Tbg*HpHbR^V293A^ and *Tbg*HpHbR^ GA369-370EG^ cell lines. Based on these results, the highly conserved amino acid substitution in the *Tbg*HpHbR at position 210 is responsible for decreased binding affinity for TLF-1.

In flow cytometry studies, a small amount of TLF-1 binding to the *Tbg*HpHbR was detected (Figure 4B). To determine whether this represented binding to the *Tbg*HpHbR or a low level of background binding we over-expressed the *Tbg*HpHbR and the *Tbb*HpHbR through ectopically expressing the HpHbR, driven by a ribosomal promoter, in the *Tbb*HpHbR^−/−^ cells. This resulted in a 15-fold increase in expression of *Tbg*HpHbR and the *Tbb*HpHbR mRNAs as measured by qRT-PCR (Table 1). Overexpression of the *Tbb*HpHbR also resulted in a large increase in TLF-1 binding (16-fold) and sensitivity to TLF-1 killing (18-fold). However, over-expression of *Tbg*HpHbR, to similar levels, had no effect on TLF-1 binding or susceptibility (Table 1). Together these results indicate that the *Tbg*HpHbR is unable to bind TLF-1 and that a single amino acid change in the *Tbg*HpHbR is sufficient to spare *T. b. gambiense* from TLF-1 killing.

**Table 1:** Over expression of *Tbb*HpHbR and *Tbg*HpHbR.

Cell Line	HpHb mRNA^1^	TLF-1 binding^2^	TLF-1 killing^3^
*T. b. brucei*	1.0	1.0	1.0
KO	N.D.	N.D.	N.D.
*Tbb*HpHbR	1.1±0.2	1.0	1.0
*Tbb*HpHbR (over-expressed)	15.8±2.0^4^	17.0±0.2^5^	17.8±2.2^6^
*Tbg*HpHbR	0.9±0.1	N.D.	N.D.
*Tbg*HpHbR (over-expressed)	11.2±4.0^4^	N.D.^5^	N.D.^6^

N.D. = Not Detectable.

1 ) determined by qRT PCR (Fig. 1B).

2 ) determined by FAC analysis (Fig. 2D).

3 ) determined by 16 hr. survival assays (Fig. 2G, 2H).

4 ) determined by qRT PCR (data not shown).

5 ) determined by FAC analysis (data not shown).

6 ) determined by 2 hr. lysis assays (data not shown).

## Discussion

Previous studies have shown that the level of HpHbR expression can influence the susceptibility of African trypanosomes to TLF-1 and human serum [4], [23]. Analysis of mRNA levels in five field isolates of group 1 *T. b. gambiense* showed that *Tbg*HpHbR expression was reduced 20-fold relative to *T. b. brucei* [23]. In addition to reduced mRNA levels, four non-synonymous substitutions present in the *Tbg*HpHbR and not in *Tbb*HpHbR were identified [23]. A more extensive analysis of HpHbR gene sequences from 67 isolates of *T. b. brucei, T. b. gambiense* group 1 and group 2 and *T. b. rhodesiense* supported these findings and further narrowed conserved substitutions in *Tbg*HpHbR. This led to the suggestion that substitution of leucine with serine at position 210 might abolish TLF-1 binding [23]–[25].

To directly test the consequence of amino acid substitutions within the *Tbg*HpHbR, on TLF-1 binding, uptake and trypanolytic activity we established a *Tbb*HpHpR^−/−^ cell line by replacement of both alleles with drug resistance markers (Figure 1A). Using this stable cell line, we tested each amino acid substitution in the *Tbg*HpHbR individually by ectopic expression (Figures 3B). By systematically changing each of the amino acid substitutions in the *Tbg*HpHbR to the most common sequence in *Tbb*HpHbR, we showed that the S210L change restores high affinity TLF-1 binding, uptake and trypanosome killing (Figure 3, 4). Based on these new findings and our previous results we propose that group 1 *T. b. gambiense* has evolved two mechanisms to avoid uptake of TLF-1. First, the abundance of HpHbR mRNA was reduced 20-fold in all group 1 *T. b. gambiense* isolates tested [23]. Secondly, as shown in the studies presented here, the *Tbg*HpHbR had reduced affinity for TLF-1 due to an amino acid substitution that was highly conserved in all members of this subgroup. It is likely that both reduced HpHbR expression and TLF-1 affinity contribute to the overall resistance of group 1 *T. b. gambiense* to TLF-1.

Recent crystallographic studies of the *T. congolense* HpHbR have allowed a detailed structural analysis of the trypanosome HpHbR [26]. These studies revealed a hydrophobic core head domain predicted to be important in receptor-ligand interaction and further predicted that the hydrophobic core of the ligand-binding domain would be disrupted by the S210L substitution described in Figure 3. We found that addition of a HA-epitope within the disrupted head domain of the *Tbg*HpHbR was accessible for antibody detection (Figure 2B). In contrast, the HA-epitope, in the stabilized head domain of the *Tbb*HpHbR, was inaccessible to antibody binding yet retained TLF-1 binding and facilitated killing (unpublished data). The *in vivo* binding results presented in Figures 3 and 4, were also consistent with SPR binding assays with recombinant HpHbR, which showed that the leucine to serine substitution significantly reduced TLF-1 and HpHb binding to the HpHbR [26].

A potentially important difference in TLF-1 binding was revealed in the *in vitro* binding studies with recombinant HpHbR [26] and the *in vivo* studies reported here (Figure 3). The SPR binding results showed a striking difference in the affinity for TLF-1 and HpHb for the *Tbb*HpHbR (5–10 µM and 4.5 nM respectively) [26]. This is inconsistent with our findings showing that TLF-1, when saturated with bound Hb, binds with a similar affinity as HpHb to the *Tbb*HpHbR (Figure 3). The relatively low affinity binding of TLF-1 may result from sub-saturating levels of Hb in the TLF-1 samples used in their studies. Alternatively, the higher affinity measured *in vivo* may reflect a role for secondary binding proteins on the trypanosome surface that increase the binding affinity of the heterogeneous TLF-1 particles [30].

Other mechanisms of resistance contribute to human infectivity by group 1 *T. b. gambiense* since TLF-2 kills HpHbR deficient *T. b. brucei* lines, albeit at concentrations approximately 200-fold higher than needed to kill WT *T. b. brucei* [4]. It is possible that group 1 and group 2 *T. b. gambiense* share common, HpHbR independent, mechanisms of resistance. Unlike group 1 *T. b. gambiense*, the sequence of the HpHbR gene in subgroup 2 more closely resembles that of *T. b. brucei*. Critically, the group 2 *T. b. gambiense* HpHbR has a leucine at position 210 [24], [25]. TLF-1 binding, uptake and trafficking to the lysosome of group 2 *T. b. gambiense* also more closely resemble the *Tbb*HpHbR [24].

The HpHbR has been proposed to be an essential nutrient receptor in African trypanosomes functioning in hemoglobin scavenging in these heme auxotrophs [14]. The near wild type growth rate of *T. b. brucei* HpHbR^−/−^ cell line showed that the receptor was not essential for survival *in vitro* (Figure 1). Furthermore, this suggests that heme scavenging, by the HpHbR, may not be necessary in bloodstream African trypanosomes. An attractive alternative is that the *T. b. brucei* HpHbR^−/−^ cell lines have other mechanisms for heme uptake that can compensate for the loss of the HpHbR under *in vitro* growth conditions. Recently, a heme transporter has been described in Leishmania that is partially localized to the plasma membrane suggesting that heme may be transported into kinetoplastids in the absence of the HpHbR [31].

It is not surprising that group 1 *T. b. gambiense* has evolved diverse mechanisms for protection against TLF-1 and 2. These parasites have a long and intimate involvement with the human host. Largely lacking wild game or domesticated animal reservoirs, these parasites have had ample opportunities to develop both redundant and augmenting mechanisms of resistance. It is likely that the observed reduced expression and loss of function substitution to the HpHbR gene in group 1 *T. b. gambiense*, though seemly redundant processes, heightens the collective resistance of these cells to the more complex assault by the human innate immune systems. Group 2 *T. b. gambiense* is genetically more diverse than group 1 and has evolved a novel HpHbR independent mechanism for inhibition of TLF-1 killing [24]. Since group 2 *T. b. gambiense* express a functional HpHbR, resistance requires inhibition of TLF-1 killing. It is appealing to speculate that group 1 *T. b. gambiense* may share this mechanism but its effect on TLF-1 killing is largely masked by reduced TLF-1 uptake.

## Supporting Information

Figure S1 Genomic PCR analysis. HpHbR-specific primer PCR analyzed the presence of DNA for the HpHbR in both wild type *T. b. brucei* (WT) and *Tbb*HpHbR^−/−^ (KO). Transcript presence is indicated by PCR band appearance with β-tubulin used as the loading control.
(TIF)

Figure S2 Saturation of TLF-1 binding by Hb. To ensure that all TLF-1 was saturated with Hb in the competition binding assays, Hb was added to TLF-1 (3 nM constant) in increasing concentrations. The percentage of maximum binding measured from FAC analysis is plotted versus the concentration of Hb.
(TIF)

Figure S3 Comparative binding curve for TLF-1 measured by fluorescence microscopy and FAC analysis. For both analyses a Kd of 4.06±0.08 nM (microscopy) and 3.42±0.52 nM (FAC analysis) was determined.
(TIF)

Methods S1 Supporting Methods.
(DOCX)

Table S1 Oligonucleotides used in (RT) PCR experiments.
(TIF)

Table S2 Relative HpHbR mRNA levels.
(TIF)
