# Supplementary figures and images for: A Single Amino Acid Substitution in the Group 1 Trypanosoma brucei gambiense Haptoglobin-Hemoglobin Receptor Abolishes TLF-1 Binding
Source: PLoS Pathog. 2013 Apr 18;9(4):e1003317. doi: 10.1371/journal.ppat.1003317 (PMC3630162; doi:10.1371/journal.ppat.1003317)

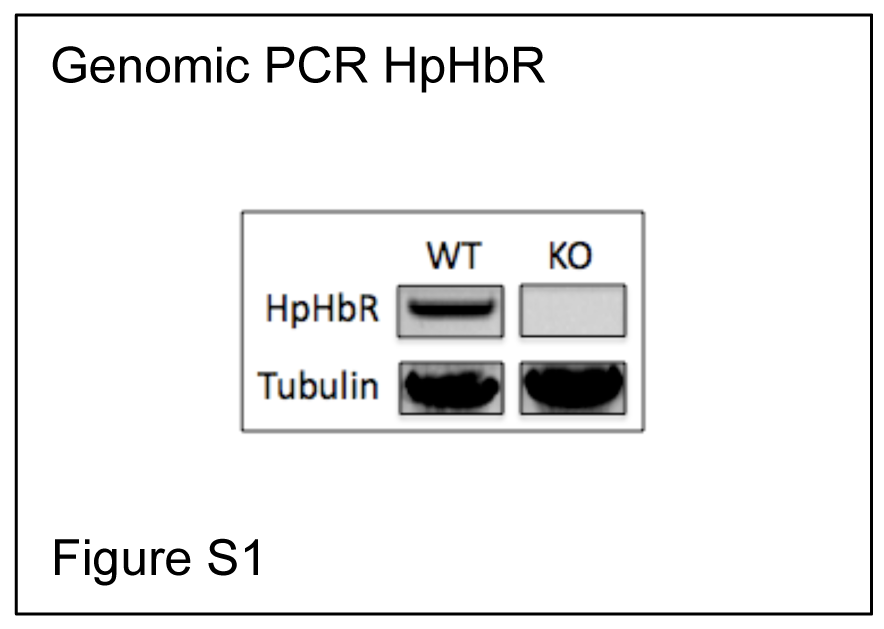

Supplement: Figure S1 — Genomic PCR analysis. HpHbR-specific primer PCR analyzed the presence of DNA for the HpHbR in both wild type T. b. brucei (WT) and TbbHpHbR−/− (KO). Transcript presence is indicated by PCR band appearance with β-tubulin used as the loading control. (TIF) [file ppat.1003317.s001.tif]

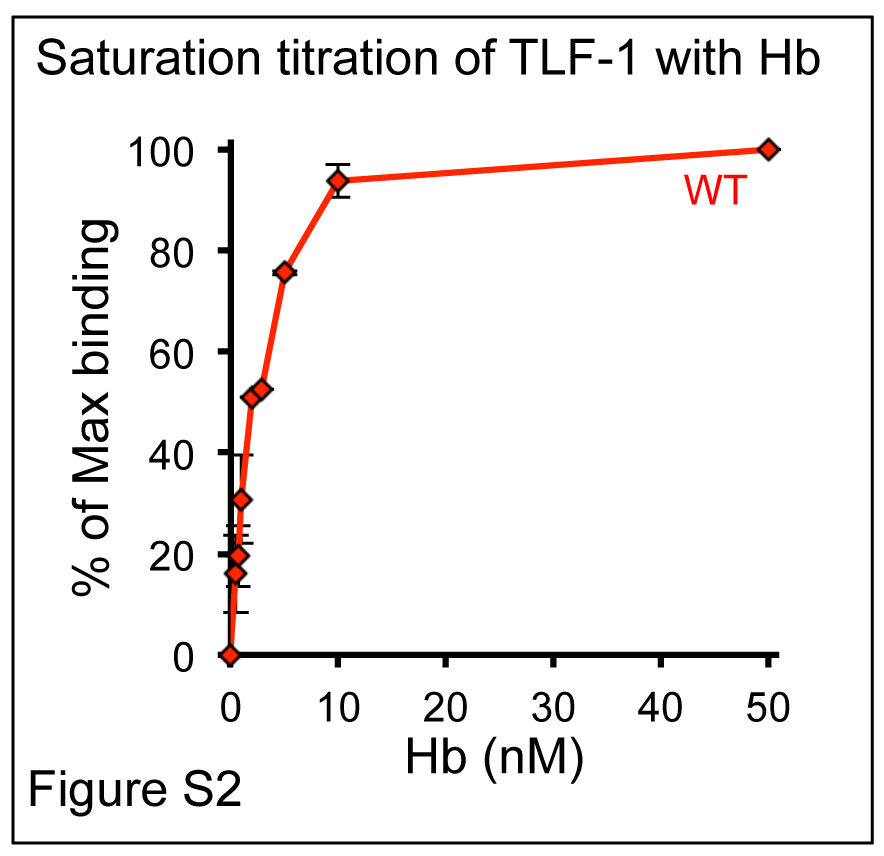

Supplement: Figure S2 — Saturation of TLF-1 binding by Hb. To ensure that all TLF-1 was saturated with Hb in the competition binding assays, Hb was added to TLF-1 (3 nM constant) in increasing concentrations. The percentage of maximum binding measured from FAC analysis is plotted versus the concentration of Hb. (TIF) [file ppat.1003317.s002.tif]

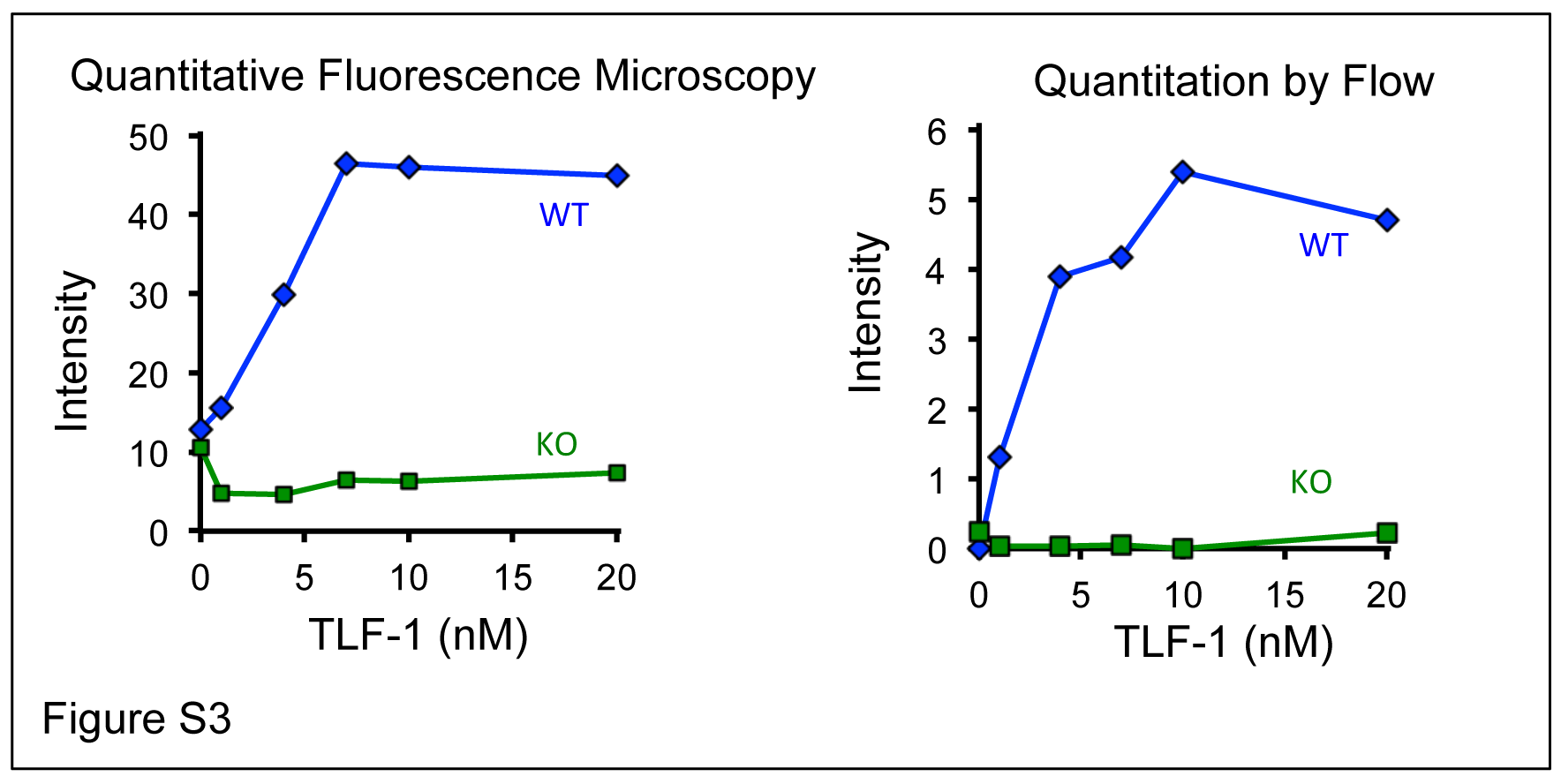

Supplement: Figure S3 — Comparative binding curve for TLF-1 measured by fluorescence microscopy and FAC analysis. For both analyses a Kd of 4.06±0.08 nM (microscopy) and 3.42±0.52 nM (FAC analysis) was determined. (TIF) [file ppat.1003317.s003.tif]

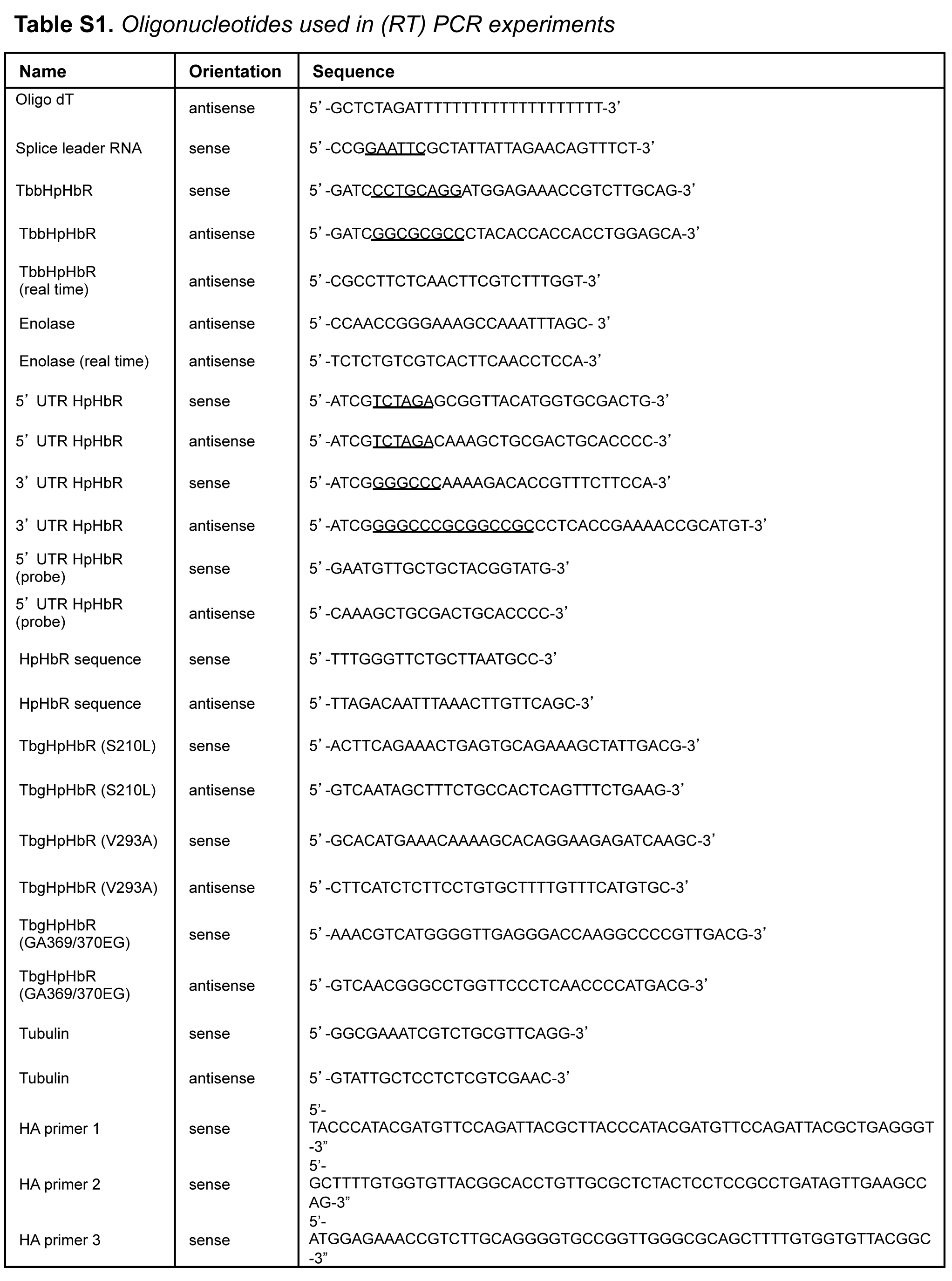

Supplement: Table S1 — Oligonucleotides used in (RT) PCR experiments. (TIF) [file ppat.1003317.s005.tif]

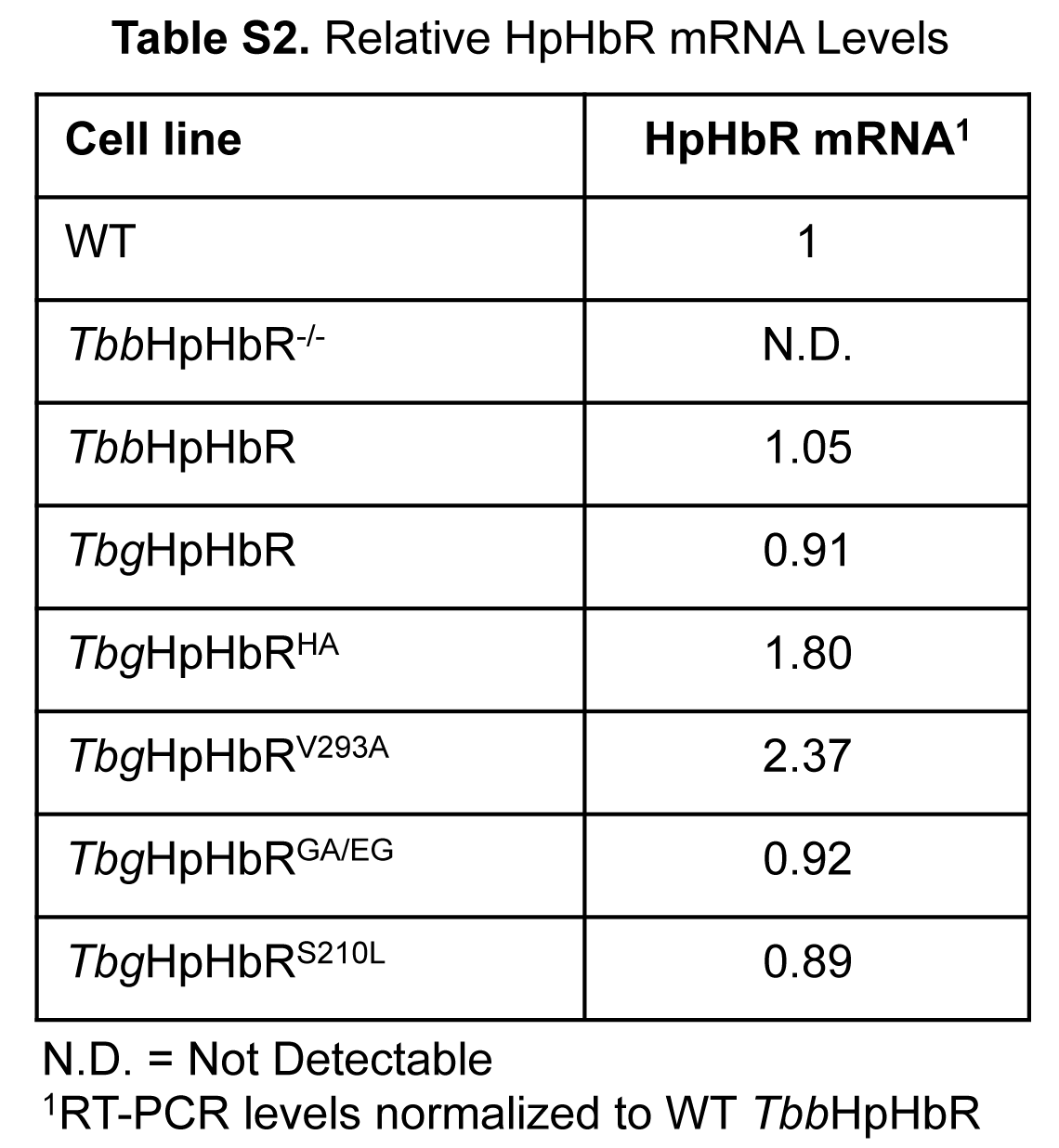

Supplement: Table S2 — Relative HpHbR mRNA levels. (TIF) [file ppat.1003317.s006.tif]
