# Supplementary material for: A Single Amino Acid Substitution in the Group 1 Trypanosoma brucei gambiense Haptoglobin-Hemoglobin Receptor Abolishes TLF-1 Binding
Source: PLoS Pathog. 2013 Apr 18;9(4):e1003317. doi: 10.1371/journal.ppat.1003317 (PMC3630162; doi:10.1371/journal.ppat.1003317)
Supplement: Methods S1 — Supporting Methods. (DOCX) [file ppat.1003317.s004.docx]

**Methods S1**

*Genomic PCR Analysis*: Genomic PCR of the HpHbR in WT and KO cell lines. 1x10^7^ cells were collected and genomic DNA was extracted**.** Primers used for amplification in these analyses are in Supplemental Table 1. PCR amplification of genomic DNA was carried out with primers specific for the open reading frame of the HpHbR and analyzed on a 0.8% agarose gel. PCR conditions were: one cycle of 10 minutes at 95°C, 25 cycles at 95°C for one minute, 56°C for one minute and 72°C for two minutes. This was followed by 72°C for ten minutes, ending with a hold 10°C. Genomic PCR analysis on the same DNA with tubulin primers was used as a control.

*TLF-1 Hb titrations:* Cells were collected, washed and resuspended (1x10^7^/ml) in ice-cold HMI-9 supplemented with 1% BSA, 1% glucose then placed in a thermocycler at 3°C for 10 minutes. Increasing concentrations of Hb were added to Alexa-488 TLF-1 (3 nM constant) and were allowed to incubate with wild type *T. b. brucei* cells at 3°C for two hours. The cells were transferred to ice, washed two times with ice-cold 1X PBS and analyzed by Cyan cytometer and FlowJo software.

*Quantitative binding analysis:* Wild type *T. b. brucei* and *Tbb*HpHbR^-/-^ (KO) cells were each subjected to the 3°C binding protocol previously described. Upon completion of incubation, cell aliquots were analyzed by both fluorescence microscopy and flow cytometry as described. Fluorescence intensity values from AxioVision v4.6 and the median intensity values from FAC analysis were plotted versus TLF-1 concentrations. All binding analyses were done in triplicate.
